# Biofilm development in three-dimensional models infected with *Trichophyton rubrum*

**DOI:** 10.1128/spectrum.01087-25

**Published:** 2025-08-29

**Authors:** Matheus Bordoy Mendonça, Ana Karla Lima Freire Cabral, Bruno Bulgarelli Adorno Arantes, Rafaela Cristine dos Santos, Kaila Petronila Medina-Alarcón, Kelvin Sousa dos Santos, Marcos William De Lima Gualque, Lígia de Souza Fernandes, Jenyffie Araujo Belizario, Thais Yume Toriy Fuzinaga, Camila Martins Kawakami, Ana Julia Pasuch Gluzezak, Luis R. Martinez, Lorena Rigo Gaspar Cordeiro, Andrei Moroz, Ana Marisa Fusco Almeida, Maria José Soares Mendes-Giannini

**Affiliations:** 1 Department of Clinical Analysis, School of Pharmaceutical Sciences, São Paulo State University UNESP 153998 , Araraquara, Brazil; 2 Laboratory of Medical Mycology, Federal University of Amazonas-UFAM 67892, , Manaus, Brazil; 3 Department of Pharmaceutical Sciences, University of Sao Paulo 42495 , Ribeirao Preto, Brazil; 4 Department of Oral Biology, University of Florida College of Dentistry 164889, Gainesville, Florida, USA; 5 Emerging Pathogens Institute, University of Florida 3463, Gainesville, Florida, USA; 6 Center for Immunology and Transplantation, University of Florida 3463, Gainesville, Florida, USA; 7 McKnight Brain Institute, University of Florida 3463, Gainesville, Florida, USA; 8 Center for Translational Research in Neurodegenerative Disease, University of Florida 3463, Gainesville, Florida, USA; Brown University, Providence, Rhode Island, USA

**Keywords:** biofilms, dermatophytes, models of infection, *Trichophyton rubrum*

## Abstract

Dermatophytes are keratinophilic filamentous fungi that cause dermatophytosis, and the main etiological agents can be anthropophilic and zoophilic. Several virulence factors are involved in the pathogenesis of dermatophytosis, including the formation of fungal biofilms. In this context, three-dimensional (3D) models, such as spheroids and reconstructed human skin (RHS), have gained prominence, as they more accurately emulate fungus-host interactions, closely resembling physiological conditions. Therefore, the present study investigated the biofilm formation of *Trichophyton rubrum* in these 3D models using confocal microscopy, scanning electron microscopy, and relative gene expression analysis via real-time PCR. Microscopic analyses revealed the colonization of the spheroid and 3D skin model surface by *T. rubrum*, with characteristics indicative of biofilm formation. The gene expression analysis of the infected 3D skin model revealed an exacerbated expression of *Mep5*, which encodes a metalloprotease in *T. rubrum*, known for its keratinolytic activity. This study demonstrates biofilm formation and protease gene expression during dermatophyte infections using 3D models that contribute to understanding the mechanisms of *T. rubrum* infection and support the ongoing search for the development of new drugs to treat dermatophytosis.

**IMPORTANCE:**

Fungal skin infections, particularly those caused by dermatophytes like *Trichophyton rubrum*, are widespread and often neglected, resulting in significant health burdens and the development of antifungal resistance due to their virulence factors, such as biofilm formation. Traditional *in vitro* and *ex vivo* infection models fail to mimic the human skin environment accurately, lacking key features, such as keratinization and three-dimensional (3D) configuration, which are critical for emulating *in vivo* infection conditions. The development of alternative 3D models, such as reconstructed human skin and spheroids, presents a transformative opportunity to enhance our understanding of host-parasite interactions. These models more closely replicate the structural and physiological properties of human skin, enabling the observation of fungal invasion and biofilm behavior under more realistic conditions. By supporting complex cellular communication and maintaining tissue architecture, 3D models provide a more accurate platform for studying fungal pathogenesis, ultimately paving the way for identifying new therapeutic targets and improving strategies to combat persistent and drug-resistant infections.

## INTRODUCTION

Fungal infections are often medically neglected and caused by poorly studied molds. Nevertheless, they impact over 1 billion individuals worldwide, resulting in high morbidity, mortality, and social stigma (1, 2). Superficial fungal infections are estimated to affect approximately 20–25% of the global population (3). Dermatomycoses are mainly caused by dermatophytes, other filamentous fungi, and *Candida* species (4). Dermatophytes are fungi that invade and proliferate within keratinized tissues (skin, hair, and nails), causing dermatophytosis, commonly known as "ringworm" or "tinea" (5). *Trichophyton rubrum* is the most prevalent and frequently isolated species in onychomycosis, tinea pedis, tinea cruris, and tinea corporis (6, 7).

Fungal skin infections generally occur commensally. Fungal growth is associated with superficial epidermal conditions, leading to keratin degradation and interaction with the host immune system (8). Although typically manageable, recalcitrant infections may become extensive and drug-resistant due to therapeutic failures (9–12). The pathogenic mechanisms of these fungi involve the adhesion of infective arthroconidia to keratinized surfaces via fungal cell wall glycoproteins and mannans, which facilitate rapid attachment to host tissues (13–15). Subsequently, the arthroconidia germinate, and hyphae invade the stratum corneum, digesting keratin into small peptides and amino acids. The first cells to be affected are keratinocytes (15–17), which recognize pathogens via pathogen-associated molecular patterns (PAMPs) through pattern recognition receptors (PRRs) (18–20). In contrast, dermatophytes secrete proteases that contribute to infection, particularly during the stages of skin tissue adhesion (21, 22) and invasion (23–26). The fungus synthesizes and secretes various enzymes that break down human proteins, such as keratin, and subsequently takes them up for metabolism. *T. rubrum* has 31 known genes encoding secreted proteases, an extensive arsenal for extracellular proteases, with differences in protease expression contributing to variations in ecological preferences as well as different sources of keratin (27).

Dermatophytes release endoproteases that cleave peptide bonds within polypeptides, whereas exoproteases cleave peptide bonds only at the N- or C-termini of polypeptides. Overall, multiple endoproteases have been broadly categorized into two prominent protein families: subtilisins, which are serine proteases (SEP), and fungalysin or metalloprotease (MEP). Subtilisins along with Sub1–12, from the largest group of endoproteases, followed by fungalysins Mep1–5 and deuterolysins Mep6–10 (28). These proteases have been demonstrated in some dermatophyte genera (29); however, they were conducted in planktonic cultures. Therefore, the endoprotease gene expression of *T. rubrum* biofilms must be investigated to gain a deeper understanding of its roles during infection.

The infection progresses further with the formation of biofilms, characterized as sessile microbial communities that adhere to the surface and each other by forming an extracellular polymeric matrix (30–32). As a virulence factor, biofilms offer several advantages, including an ideal configuration for nutrient absorption, antifungal protection, and cellular communication, such as quorum sensing, which contributes to infection persistence by regulating efflux pump genes (31, 33, 34). Our research group first described biofilm formation by *T. rubrum* and *T. mentagrophytes* (31). These biofilms are directly associated with antifungal therapy resistance and linked to the high recurrence rates of dermatophytosis (32, 35, 36).

Accordingly, various *in vitro* and *in vivo* infection models have been developed, including monolayer cultures of epidermal cells and nail or hair samples, and *ex vivo* infection models using human skin explants (8, 24, 25, 37, 38). However, these models exhibit severe limitations, as they lack live keratinocytes, which prevents assessing possible host responses to infection. In contrast, monolayers of cultured keratinocytes lack keratinization, a necessary process for analyzing dermatophytosis and its pathogenesis. In addition, human skin explants are limited by the availability and variability between samples. Recently, skin equivalents have been employed to overcome these limitations and appear promising for mimicking disease lesions and testing antifungal efficacy (29, 39–41). These studies have investigated various infection conditions; however, no comparative studies compare planktonic and biofilm-derived cells or biofilm development across different models. Thus, new models that enable robust, faster, and large-scale studies capable of mimicking biofilm formation *in vivo* are urgently needed to deepen our understanding of fungal pathogen-host interactions.

Three-dimensional (3D) cell culture models comprise multicellular layers surrounded by an extracellular matrix, which provides protection, facilitates cellular communication, and maintains tissue structure. Artificial skin that provides this critical 3D structure has been reconstructed *in vitro*. These models preserve physiological characteristics comparable to *in vivo* conditions and offer greater ease for real-time experimentation through various tools for studying fungal-host interactions, thereby considerably reducing the number of animals needed for research (42–44). For two decades, the role of biofilms in human medicine has gained attention. Yet, the need remains to demonstrate biofilm formation in *in vivo* or *ex vivo* models and identify infection biomarkers. Therefore, developing 3D skin culture models with dermatophytes in mono- or polymicrobial biofilm form, along with gene expression and correlation with pathogenesis, is crucial for advancing research on new antifungal targets, given that biofilms significantly contribute to antimicrobial resistance.

To further comprehend the interaction between host and parasite, this study analyzed *T. rubrum* biofilm formation in two 3D alternative models, reconstructed human skin (RHS) and keratinocyte spheroids, in comparison to *in vitro* culture. Well-established virulence genes were also selected to evaluate the infectivity of the fungus under conditions that more closely resemble clinical infections, thereby contributing to the understanding of mechanisms employed during infection and possibly informing the selection of targets for future therapies against dermatophytosis.

## MATERIALS AND METHODS

### *In vitro* formation of *T. rubrum* monospecies biofilm from planktonic cells

The fungal strain used was *T. rubrum* ATCC MYA-4438 from the mycological collection of the Mycology Laboratory at the Proteomics Center of São Paulo State University "Júlio de Mesquita Filho," Araraquara campus. The strain was identified by observing its micromorphology using a Primovert inverted optical Microscope (Zeiss).

The fungal strain was reactivated in a malt extract agar medium supplemented with a keratin source to promote biofilm formation. The culture was then inoculated onto potato dextrose agar medium. Both subcultures were maintained in a biochemical oxygen demand (BOD) incubator at 28°C for 7 days to induce conidia formation (32). After incubation, inoculum was prepared in 5 mL of 0.85% saline solution. The supernatant containing the conidia was collected into a new tube, and a 100 µL aliquot was taken and diluted in 900 µL (1:10) of 0.85% saline solution to analyze the conidia concentration in the inoculum using a hemocytometer. Based on the calculated concentration, a sufficient volume of culture medium was added to dilute the inoculum to a concentration of 1 × 10^6^ CFU/mL (32). The plate was then incubated at 37°C for up to 96 h. The biofilm was formed by more closely simulating infection conditions, incubating reproductive structures, the microconidia inoculum, at body temperature (37°C), in opposition to the planktonic culture derived from hyphae and conidia and incubated at room temperature (28°C) (32).

### Biofilm characterization by crystal violet staining and XTT reduction assay

We characterized the biofilm formation by quantifying biomass and metabolic activity using crystal violet staining and XTT reduction assay. To ensure that *T. rubrum* could form biofilms in the media used for the infection models, two different culture media, namely, RPMI 1640 medium with L-glutamine with no bicarbonate and with phenol red as a pH indicator (Gibco) and buffered with MOPS -[3-(N-morpholino) propanesulfonic acid] (Sigma-Aldrich) and 10% fetal bovine serum (FBS) (Sigma-Aldrich) and Dulbecco’s Modified Eagle Medium (Gibco) (low-glucose) with 10% FBS (Sigma), were evaluated. The inocula were prepared, and their concentration was adjusted to 1 × 10^6^ CFU/mL in both RPMI and DMEM. Next, 200 µL of the adjusted inoculum was added to each well of 96-well microplates (Kasvi) and incubated at 37°C for 96 h with no agitation (45).

The culture medium was removed from the plates, and the wells were washed twice with phosphate-buffered saline (PBS). After drying at room temperature, 200 µL of 0.1% crystal violet was added to the wells for 20 min, then removed, and the wells were washed with water or until excess staining was removed. The biofilms were then decolorized by adding 200 µL of 95% ethanol, and the wells were homogenized until the crystal violet was completely solubilized. The contents were transferred to a new microplate for spectrophotometric reading at a wavelength of 570 nm. This procedure was repeated on 24, 72, and 96 h post-biofilm formation incubation (31, 32).

For the XTT reduction assay, the culture media were removed from the wells and washed with 200 µL of PBS. Subsequently, 54 µL of a solution containing 50 µL of XTT and 4 µL of menadione were added to each well. The plates were sealed, protected from light, and incubated at 37°C for 3 h. After this time, a spectrophotometric reading was taken at a wavelength of 490 nm. This experiment was conducted simultaneously with crystal violet staining and repeated at 24, 72, and 96 h post-biofilm formation (31, 32).

### Assembly and infection of 3D skin model

The RHS model was assembled from primary cultures of keratinocytes and fibroblasts isolated from foreskin samples obtained from individuals aged up to 15 years in collaboration with Prof. Dr. Lorena Gaspar Cordeiro at the University of São Paulo (USP). The samples were used following approval by the Human Research Ethics Committee of the School of Pharmaceutical Sciences at Ribeirão Preto—USP, and informed consent was obtained following the Declaration of Helsinki and approved by the Research Ethics Committee (Resolution No. 466/12) (CAAE 85813524.1.0000.5403). The models were consistently used in pools of three donors to minimize the differences related to genetic variability among donors. The RHS was cultured in trans-well inserts (ThinCert, Greiner) in 24-well plates, as described by Spagolla et al. (46). After 6 days of cultivation in the air-liquid interface, the RHS models were preliminarily infected with a *T. rubrum* conidia solution at a concentration of 1 × 10⁶ and incubated at 37°C and 5% CO_2_ for 96 h to allow for fungal biofilm development (32).

### Assays for the 3D spheroid model formation and infection

Spontaneously transformed human keratinocyte cell culture (HaCaT) was used for the spheroid assembly. The cells were cultured in DMEM (Gibco) supplemented with 10% FBS and incubated at 37°C and 5% CO_2_ for 96 h. Following thawing, the cell line was incubated until it reached 80% confluence, treated with trypsin, and then transferred to a new culture flask. Before experimental assays, cells underwent a post-thaw passage to enhance cell-cell and cell-matrix interactions (47).

The 3D cellular spheroid model was developed as described by Friedrich et al. (47), with minor modifications. Cells were set in 96-well microplates (Kasvi) coated with 50 µL of agarose (LCG) dissolved in sterile distilled water at a concentration of 1.5%. The agarose was autoclaved and maintained in a water bath at 60°C to prevent gelling, after which it was transferred to the plates in its liquid form.

Spheroid formation was tested at various cell concentrations, ranging from 1 × 10³ to 6 × 10⁴ cells/well in DMEM medium supplemented with 10% FBS. The metabolic activity was measured by resazurin reduction assay. At 0-, 4-, and 7-day formation times, 20 µL of 50 µM resazurin solution (Sigma-Aldrich, Milano, Italy) was added to the wells and incubated under standard conditions for 3.5 h. Absorbance was measured at wavelengths of 570 and 600 nm.

After 96 h of formation, images were taken, and the radius and diameter were verified. The sphericity index was then calculated using ImageJ software (48). The cell suspensions were then transferred to the agarose-coated wells and incubated at 37°C and 5% CO_₂_ for 96 h to form spheroids with diameters between 370 and 500 µm (47–50). Spheroids were individually transferred to a new 96-well microplate, and 200 µL of a *T. rubrum* conidia suspension at a concentration of 1 × 10⁶ cells in DMEM with 10% FBS was added to each well. The plate was incubated at 37°C and 5% CO₂ for 72 h to allow for fungal biofilm formation (32).

### Confocal microscopy (CM) of fungal biofilm in three-dimensional spheroid model

To assess the interaction between *T. rubrum* and HaCaT cells, matured spheroids were infected and analyzed using CM. The infection period was 72 h, aligning with the time required for *in vitro* fungal biofilm formation and maturation. The infected spheroids were washed with 1× PBS solution to remove non-spheroid cell elements and then fixed with a 4% paraformaldehyde and Triton X-100 solution (Sigma) (48). Subsequently, the spheroids were washed with 1× PBS solution and transferred to microtubes, where the fungal structures were stained with 20 µL of calcofluor white (CW) in 1,000 µL of 1× PBS for 5 min. The staining was removed by repeated washing with 1× PBS, and the keratinocytes were then stained with a solution of 5 µL fluorescein isothiocyanate (FITC) in 950 µL of 1× PBS for 20 min. The second stain was removed by washing with 1× PBS, and the infected, stained spheroids were transferred to a new 96-well microplate (50). Alternatively, bisbenzinamide (Hoechst 33342) was also used to assess fungal development alongside the spheroid. Then, the LIVE/DEAD Kit (Applied Biosystems, Thermo Fisher) was used to assess the viability of the keratinocytes within the spheroids, where dead cells stain green (SYTOX-Green), and live cells stain red (Resazurin-C12), following the manufacturer’s protocol. The infected spheroid samples were analyzed using a confocal laser scanning microscope (CLSM; Carl Zeiss LSM 800 with Airyscan) in three channels, namely, DAPI, FITC, and rhodamine, with excitation-emission values of 350–460, 500–520, and 570–590, respectively. The obtained images were analyzed using Zen Blue 3.2 software (Carl Zeiss, Jena, Germany), and ImageJ win64.exe software was used for further processing and in-depth analysis of the images.

### Scanning electron microscopy (SEM) of fungal biofilm

A *T. rubrum* biofilm was formed in 24-well microplates, washed with sterile 1× PBS solution, fixed by adding 800 µL of 2.5% glutaraldehyde solution, and incubated at 4°C for 1 h (30, 31, 38). The glutaraldehyde was removed, and the wells were washed and dehydrated through consecutive ethanol washes of increasing concentration (50 to 100%) at room temperature. The samples' well bottoms were cut out and placed in a vacuum chamber for gold coating, which was then metalized using a Denton Vacuum Desk V (Jeol, Moorestown, NJ, USA). The topography of the structures was assessed using SEM (Joel JSM-6610LV, Moorestown, NJ, USA). Formed structures resulting from fungal infection in the 3D RHS model and *in vitro* biofilm formation after 72 h of maturation were then analyzed and compared to controls (32).

### RSH infection models

The relative expression of the *T. rubrum* virulence genes in *in vitro* biofilm form or after infection of the 3D RHS model was evaluated using real-time PCR (qPCR) and compared to planktonic cells. Total RNA was extracted using the Illustra RNAspin Mini Kit (GE Life Sciences) according to the manufacturer’s protocol, with liquid nitrogen used for maceration during the lysis step. The concentration and purity of the RNA samples were determined using spectrophotometry on a Nanodrop 2000 device (Thermo Fisher Scientific) at absorbance wavelengths of 260 and 280 nm. Integrity was assessed using capillary electrophoresis with the Agilent 2100 Bioanalyzer (Agilent Technologies, Palo Alto, CA, USA) in the multi-user laboratory of the Genomics Center at São Paulo State University "Júlio de Mesquita Filho" Araraquara campus. The RNA was treated with a DNase I Kit (Sigma-Aldrich), and complementary DNA (cDNA) was synthesized using the High-Capacity cDNA Reverse Transcription Kit (Applied Biosystems) according to the manufacturer’s instructions. The qPCR was performed using the 7500 Real-Time PCR Instrument (Applied Biosystems, Thermo Fisher Scientific) in the multiuser laboratory of the Genomics Center at São Paulo State University "Júlio de Mesquita Filho" Araraquara campus, with the Power SYBR Green PCR Master Mix Detection System (Applied Biosystems by Thermo Fisher) (51).

For the qPCR, a total volume of 20 µL per well was used, with a mix containing 100 ng of sample cDNA and 0.5 µM of each primer. The relative expression was then calculated using the 2^−∆∆Ct^ method (51).

For the major role endoproteases play in dermatophyte infections, a member of both prominent families (subtilisins and metalloproteases) was selected as the target gene (i.e., subtilisin-like protease-5 [SBT5] and extracellular metalloprotease-5 [MEP-5]), and glyceraldehyde 3-phosphate dehydrogenase (GAPDH) was used as the endogenous control gene (28, 51, 52). Table 1 provides the sequences of the oligonucleotide primers used for each gene in the qPCR analysis.

**TABLE 1:** Primers were used to evaluate the relative gene expression of *T. rubrum* genes in biofilm-derived and planktonic cells^*a*^

Gene	5′−3′ Oligonucleotides
GAPDH	F: GGTGGTTGTGGTGGGATACT
	R: CGACCTGGGCTCCTGTTAAT
Subtilisin-like protease 5	F: TCCGCCACTCTGAATTCCAA
	R: GTGTCCGGTTCCATCAGAGT
Extracellular metalloprotease 5	F: TACCTTGCCATGAAGCTCGT
	R: CCATAGACCAGCGCAGAGAA

^*a*^ F: forward; R: reverse.

### Statistical data analysis

All assays were performed in triplicate, and the experiments were repeated three times independently. Data generated by qPCR, where multiple conditions were compared, were analyzed using a one-way analysis of variance (ANOVA). Individual comparisons at various time points were analyzed using multiple Student *t*-test analyses. All generated data were analyzed using GraphPad Prism 8.0 software. Statistical significance was set at *P*-values less than 0.05.

## RESULTS

### *T. rubrum* biofilm formation was evaluated in RPMI and DMEM by XTT reduction assay and crystal violet staining

**Fig 1:**
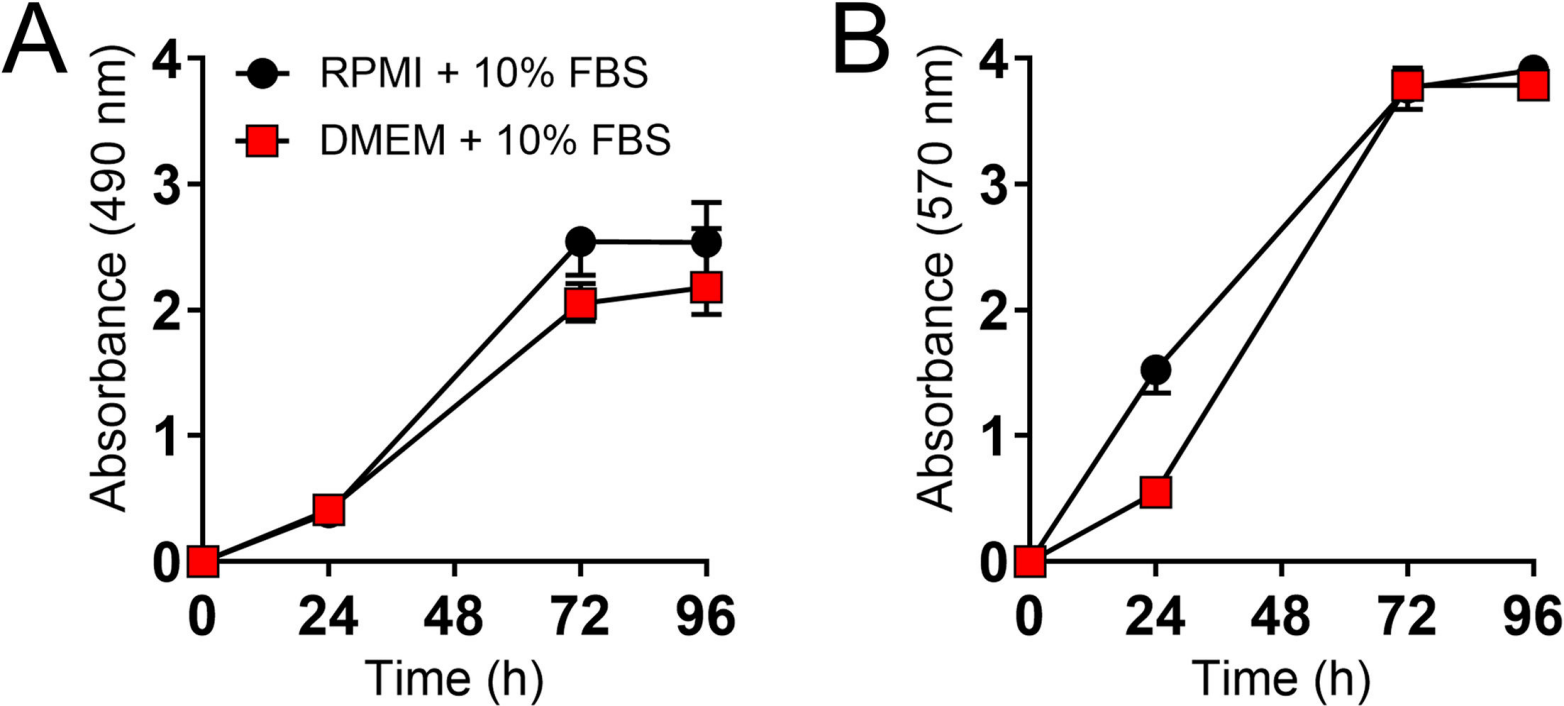
Characterization of the *T. rubrum* biofilm in two different media, RPMI and DMEM. (**A**) The metabolic activity was determined by the XTT reduction assay, and (**B**) biomass was assessed by crystal violet staining for 96 h. The statistical significance (*P* < 0.05) was evaluated using multiple Student *t*-test analyses. No difference was observed between the conditions.

Using the XTT reduction assay (Fig. 1A) to measure the metabolic activity of cells within biofilms and crystal violet staining (Fig. 1B) to assess biofilm biomass or a combination of biofilm-associated cells and extracellular matrix, we found no significant difference between RPMI and DMEM (*P* > 0.05). Both essays present biofilm formation curves in both media that are compatible with those already well described in the literature (30–35). These results indicate that *T. rubrum* can form biofilms in RPMI and DMEM media, which is ideal for our studies.

### Assembly and infection in the reconstructed human skin (RHS) model

**Fig 2:**
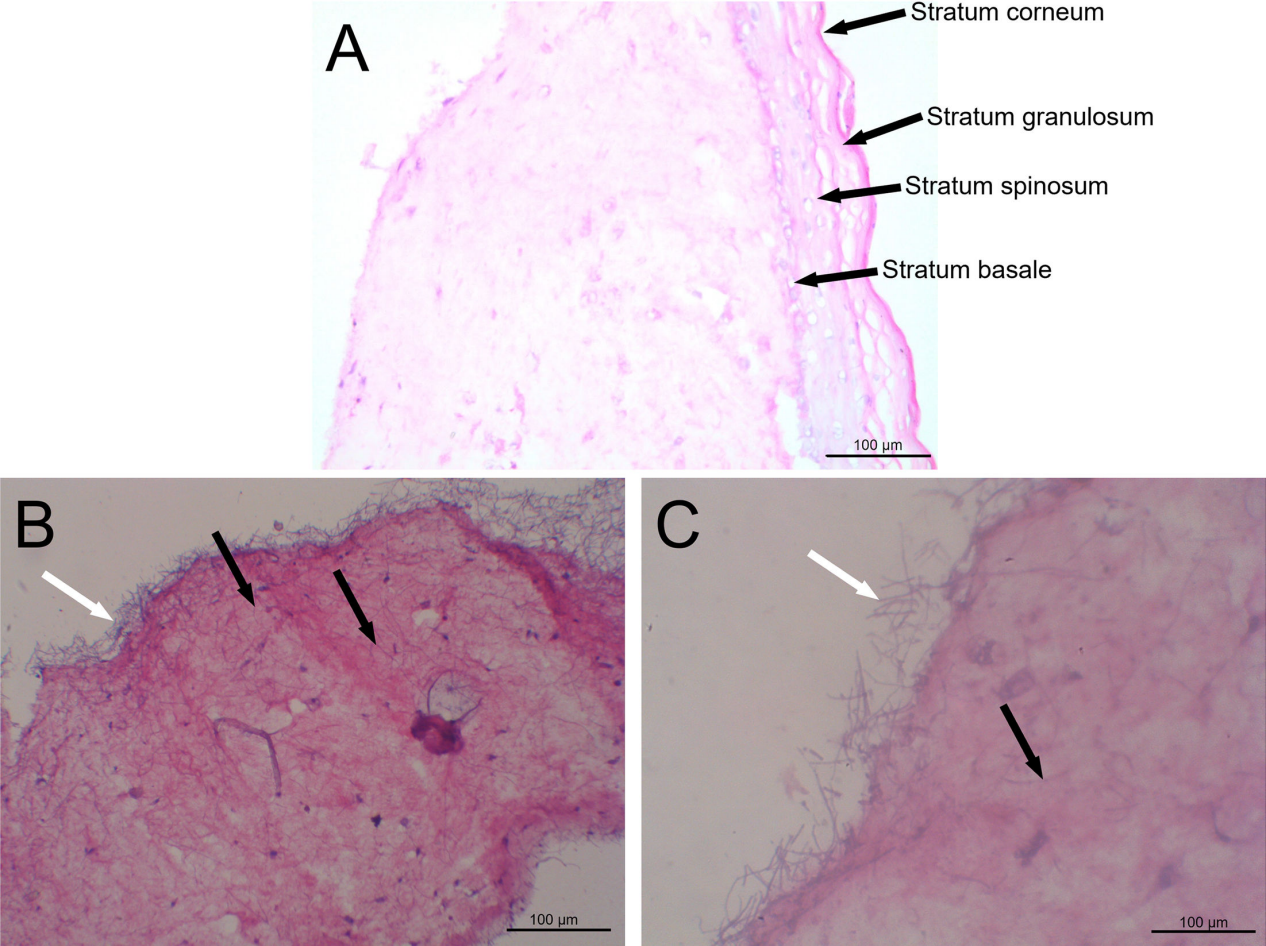
Photomicrographs of the reconstructed human skin (RHS). The histological section was stained with H&E staining for general tissue morphology. Healthy skin (A) at 40× magnification with visible epidermal stratification indicated by the arrows, and infected skin at (B) 10× and (C) 40× magnification. The white arrow indicates the presence of *T. rubrum* on the surface of the RHS. The black arrows denote apparent invasive *T. rubrum* hyphae in deeper skin tissue.

Hematoxylin-eosin (H&E) staining was employed to evaluate general tissue morphology under light microscopy. The RHS exhibited a tissue organization similar to that of native human skin, characterized by proper patterns of cellular differentiation and epidermal stratification, as well as a well-formed stratum corneum (Fig. 2A). *T. rubrum* colonization was also evaluated, showing abundant and homogeneous hyphal clusters (white arrow) in the superficial area of the skin tissue (10× image; Fig. 2B). A higher magnification (40×) of the infected tissue shows considerable accumulation of *T. rubrum* hyphae intertwined on the tissue surface (black arrow) as well as hyphal invasion of the tissue (Fig. 2C). These findings demonstrate that we can generate an RHS model as an alternative to investigate superficial skin infections, especially those caused by dermatophytes.

### Scanning electron microscopy of fungal biofilms in the 3D skin model

**Fig 3:**
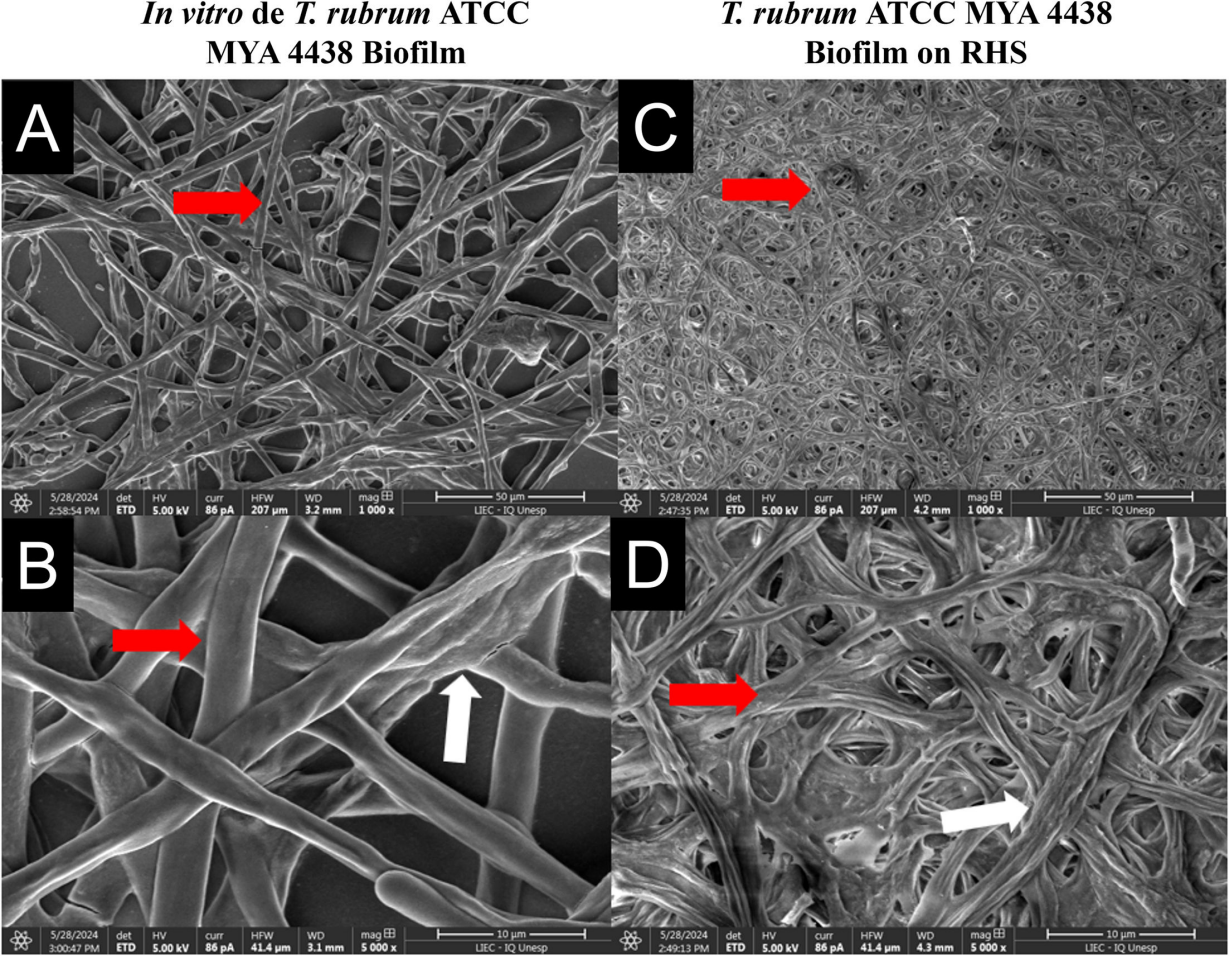
SEM of *T. rubrum* ATCC MYA 4438 biofilms in 96-well microtiter polystyrene plates (**A and B**) and the RHS-infected model (**C and D**). Magnifications of 1,000× (**A and C**) and 5,000× (**B and D**) are shown. White arrows indicate the polysaccharide material adhered to the fungal hyphae, marked by red arrows.

The fungal structures of the *T. rubrum* biofilms formed in the RHS model (Fig. 3C and D) were compared to those grown *in vitro* (Fig. 3A and B). The SEM analysis revealed that *T. rubrum* biofilms formed both *in vivo* and *in vitro*. However, the *T. rubrum* biofilms on RHS (Fig. 3C) exhibited a denser and more entangled network of elongated hyphae than those formed *in vitro* (Fig. 3A). High-magnification images showed hyphal interconnection in both abiotic (Fig. 3B) and biotic (Fig. 3D) surfaces, although only RHS biofilms demonstrated mycelial formation. *In vitro* and RHS fungal biofilms exhibited visible hyphal adhesion of extracellular polysaccharides, as indicated by the red arrows. Our findings suggest that *T. rubrum* forms more robust biofilms on the RHS model, which may mimic *in vivo* infections, making it suitable for investigating the dermatophyte biofilm formation in the laboratory.

### 3D spheroid model formation from keratinocyte origin

**Fig 4:**
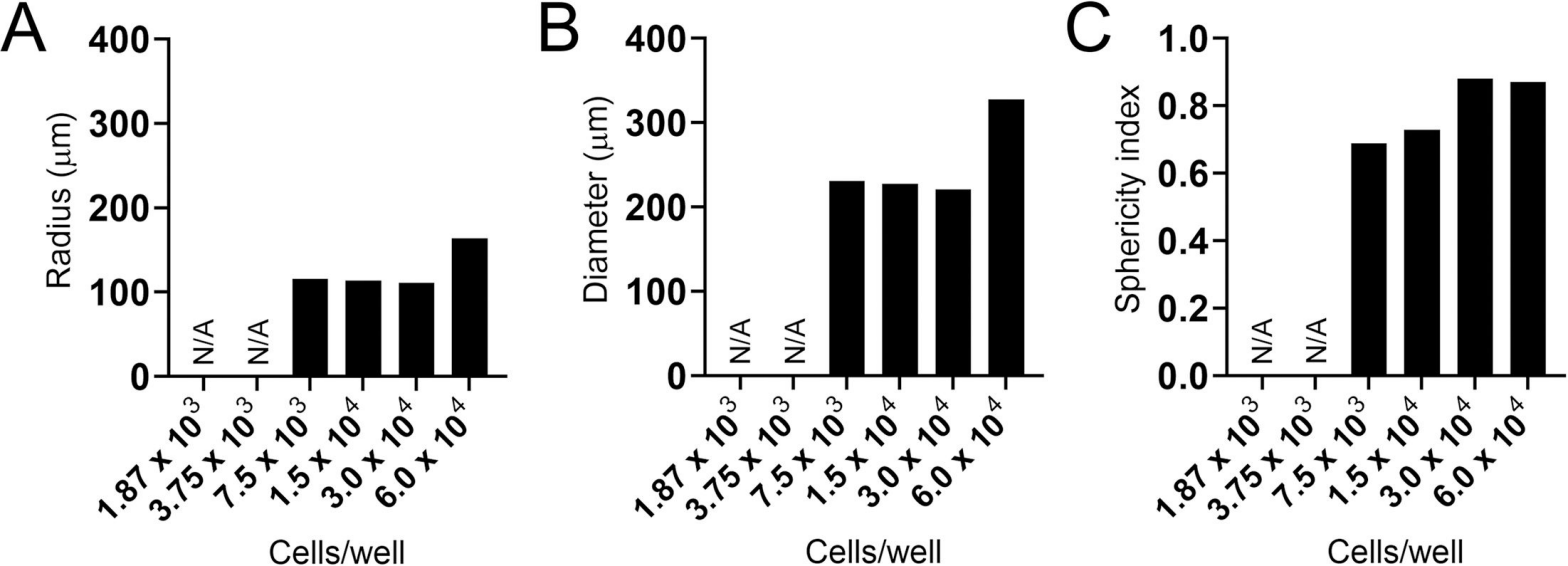
Characteristics of keratinocyte spheroids used to study the *T. rubrum* biofilm formation. (**A**) Spheroid radius, (**B**) spheroid diameter, and (**C**) sphericity index were measured as indications of the 3D spheroid model formation.

A 3D keratinocyte cell culture model was developed to study dermatophyte infections and their interactions with host cells (Fig. 4). The cell concentrations for the spheroidal structure formation using 3.0 × 10^4^ and 6.0 × 10^4^ cells/well showed the most robust and symmetrical structures, with diameters of 221.33 and 327.43 nm, respectively (Fig. 4A through C). The sphericity index reflects how closely the spheroid approximates a circle, with values near 1 indicating high circularity (Fig. 4C). Cell concentrations of 3.0 × 10^4^ and 6.0 × 10^4^ cells/well, which exhibited sphericity indices closer to unity, were selected and used in subsequent assays. Cell densities were lower than 7.5 × 10^3^ cells/well and could not form a functional spheroid model. These results demonstrate the feasibility of establishing a 3D spheroid model of skin cell origin to study the dermatophyte biofilm formation.

**Fig 5:**
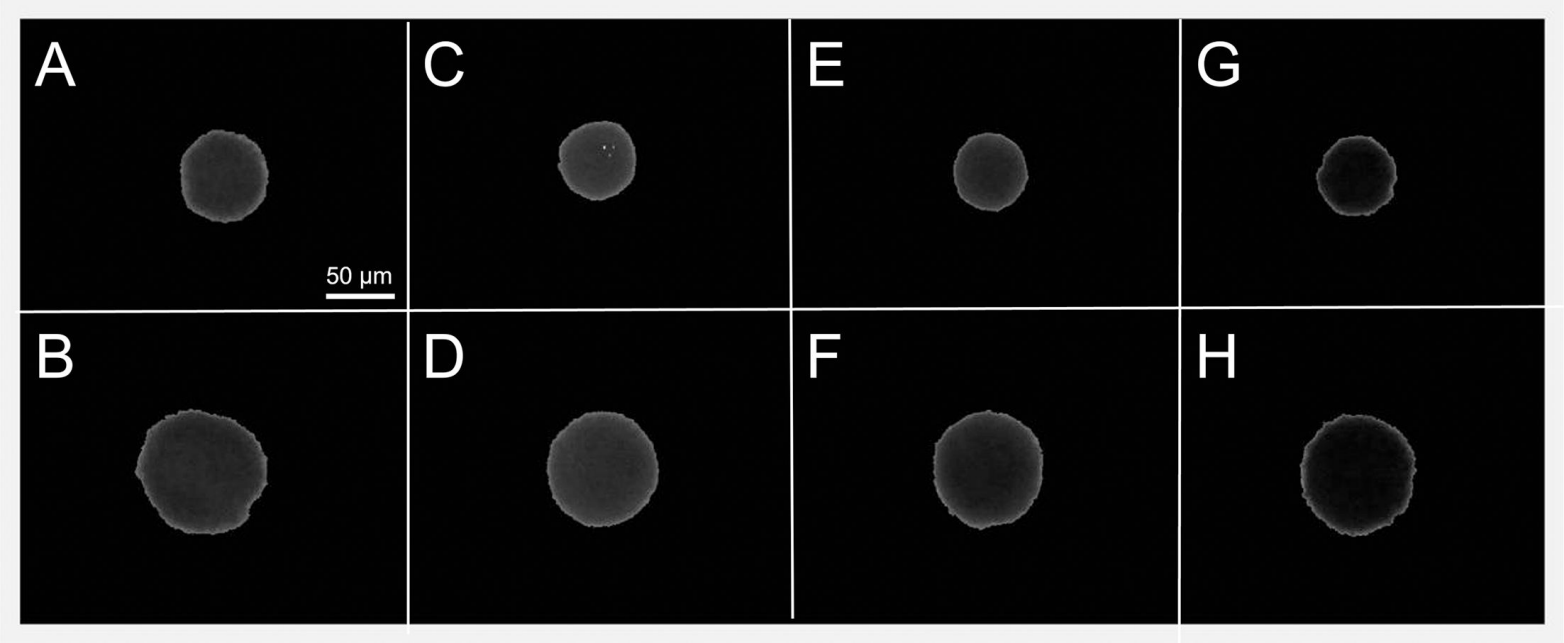
Spheroid morphology was monitored microscopically for 7 days using a resazurin reduction assay. The morphology of spheroids after 1 (**A, B**), 3 (**C, D**), 5 (**E, F**), and 7 (**G, H**) days at a cellular density of 3 × 10^4^ (upper panel) and 6 × 10^4^ cells per well (lower panel).

Spheroids formed with these cell densities were also evaluated for morphology and cell viability at 1, 4, and 7 days after formation time (Fig. 5). The morphologies of the structures were preserved throughout the evaluation period for both cell densities. The cell viability remained above 80% after 1 and 4 days of formation for both cell densities. Our data indicate that stable spheroids with high cell viability and appropriate morphology can be obtained, which are optimal for studying the *T. rubrum* biofilm formation and the host cell-fungal biofilm interactions.

### SEM of fungal biofilm in the 3D spheroid model

**Fig 6:**
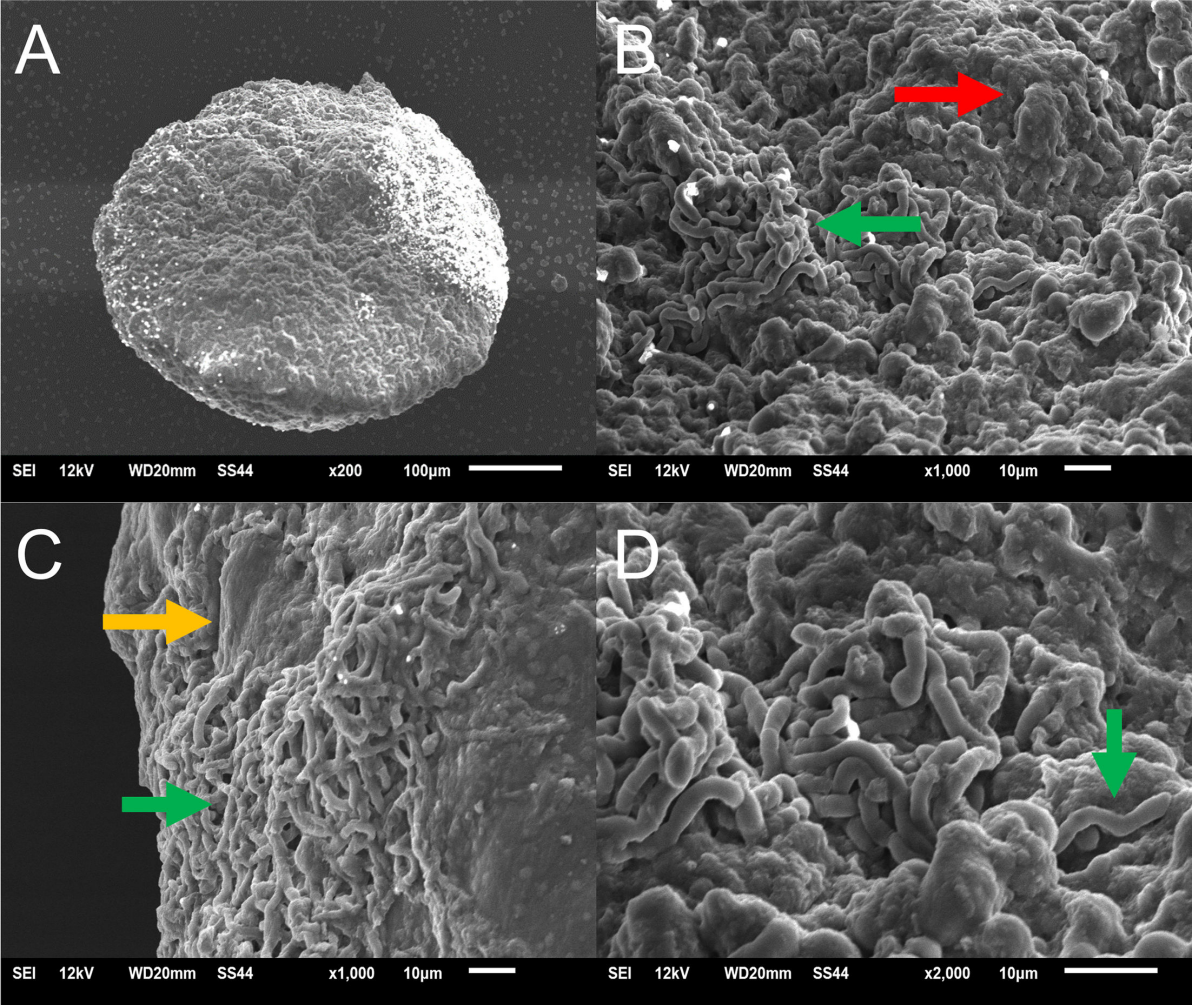
SEM images of the *ex vivo T. rubrum* biofilm formed on infected HaCaT spheroids. (**A**) A 200× magnification image reveals the general topography of the spheroid in the absence of fungus. (**B**) A 1,000× magnification image shows HaCaT cell aggregates (red arrow) and hyphae (green arrow). (**C**) A 1,000× magnification image shows the localization of the fungal extracellular matrix (yellow arrow) and hyphal aggregation (green arrow). (**D**) A 2,000× magnification image displays a close aggregation of hyphae (indicated by the green arrow).

The SEM image was captured on 7 days of culture to assess spheroid integrity and confirm the formation of a single spheroid per well. The image of HaCaT demonstrates a well-formed, complete, and rounded structure (Fig. 6A).

After the infection, a characteristic *T. rubrum* biofilm formation was observed (Fig. 6B, C and D). A compact biomass composed of a coordinated and extensive network of hyphae was observed, growing in all directions, crossing each other, and covered in some areas by a large amount of extracellular matrix, often giving the hyphae a wrinkled appearance (Fig. 6B, C and D).

### Confocal microscopy of the fungal biofilm in the 3D spheroid model

**Fig 7:**
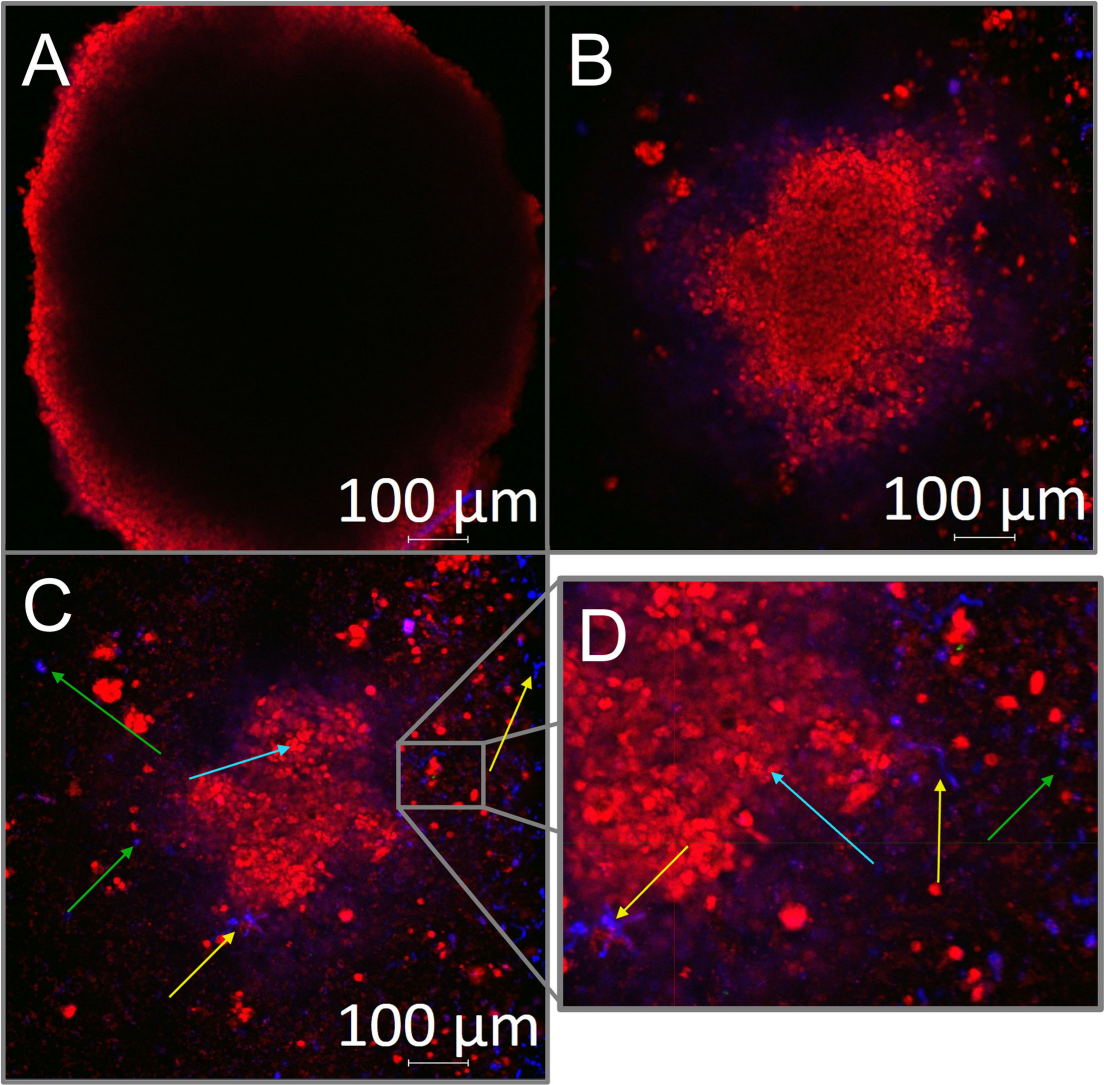
Confocal microscopy (CM) of the spheroid at 6 × 10^4^ cells/well infected with *T. rubrum*. (**A**) Full-size spheroid. (**B**) Live keratinocytes stained red with the LIVE/DEAD. (**C**) Overlay of all channels (**D**) showing a zoom-in of the infected spheroid, which highlights the fungal structures, abundance of live keratinocytes, and scarcity of dead keratinocytes. Yellow, green, blue, and white arrows indicate hyphae, conidia, live keratinocytes, and dead keratinocytes, respectively.

The CM of infected spheroids at a concentration of 6 × 10^4^ cells/well allowed us to observe that the interaction with fungal structures did not result in keratinocyte death (Fig. 7). In this experiment, dead cells emit mostly green fluorescence, while healthy, metabolically active cells emit mostly red fluorescence. A separate channel observation enabled the visualization of different aspects of spheroid formation and infection, such as its full size and shape on deeper channel (Fig. 7A). We observed an abundance of live keratinocytes (blue arrows; Fig. 7B) and their interaction with the fungus (Fig. 7C and D), with developed hyphae (yellow arrows) and conidia (green arrows) and very few dead keratinocytes (white arrows) evident. Observing an overlay of red and blue fluorescence was also possible, indicating the interaction between the fungal and keratinocyte cells.

**Fig 8:**
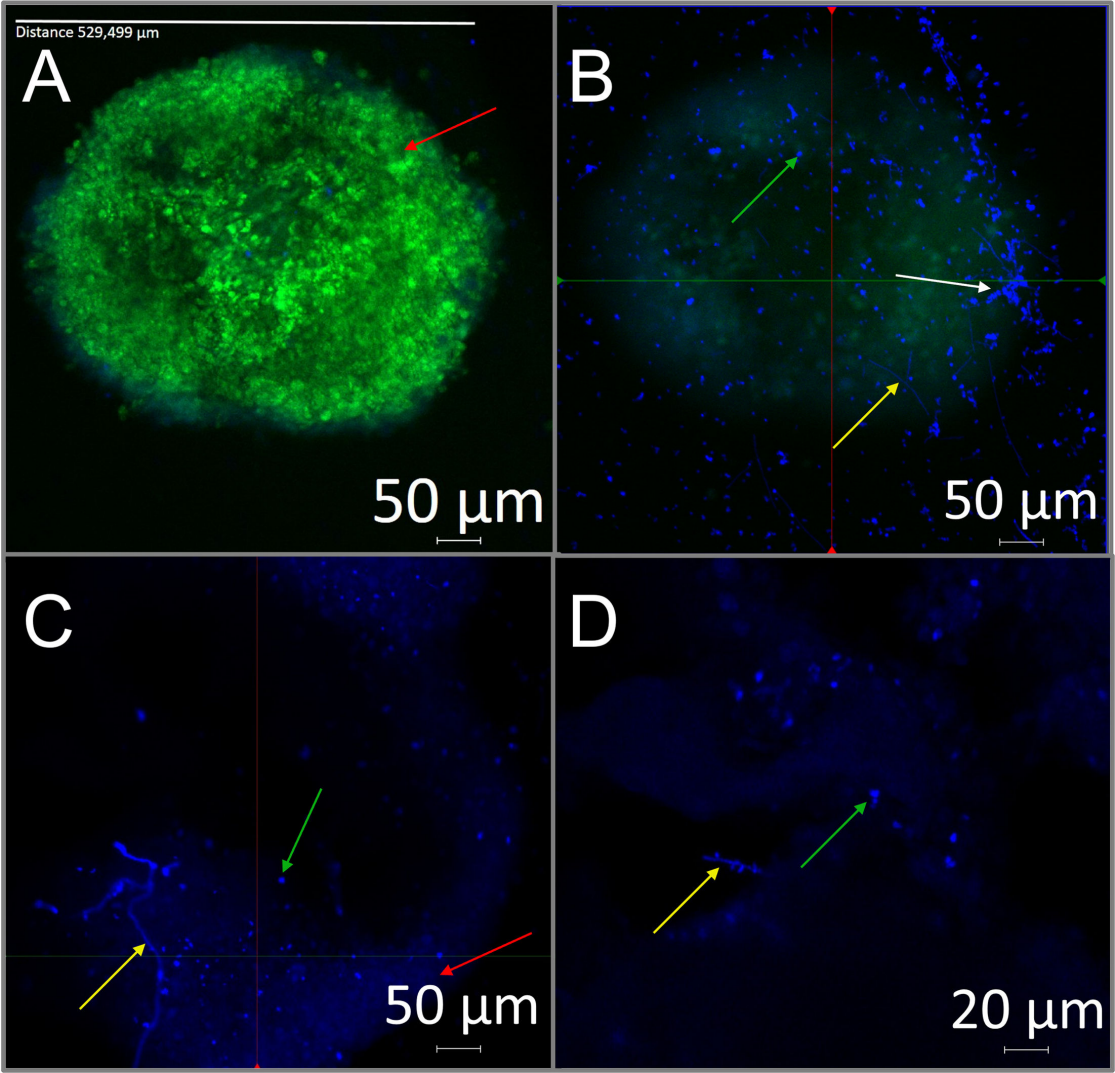
CM of a 3 × 10^4^ cells/well infected with *T. rubrum*. (**A**) On the spheroid plane. (**B**) Below the spheroid plane, a cluster of fungal cells, hyphae, and fungal conidia are visible. Keratinocytes were stained with FITC (green), and *T. rubrum* was stained with calcofluor white (blue). (**C**) Calcofluor white staining highlighted the fungal structures formed during spheroid infection. (**D**) A high-magnification image shows the fungal structures. The red, green, yellow, and white arrows indicate the keratinocyte spheroid, conidia, hyphae, and a fungal cell cluster, respectively.

The fungal structure formation was analyzed after the *T. rubrum* infection of spheroids using green fluorescence staining for keratinocytes and blue fluorescence staining for fungi (Fig. 8). Fungal development on and around the spheroid was evident (Fig. 8A), characterized by hyphal growth (yellow arrows), a significant presence of conidia (green arrows), and an aggregate of fungal cells (white arrows; Fig. 8B through D), suggesting biofilm formation. The fungal structures developed below and around the spheroid, indicating a colonization of the model by *T. rubrum*.

Our data demonstrate that we can generate 3D keratinocyte spheroids to study host cell-dermatophyte interactions.

### Real-time PCR of the *T. rubrum* gene expression in planktonic, biofilm, and reconstructed skin infection models

**Fig 9:**
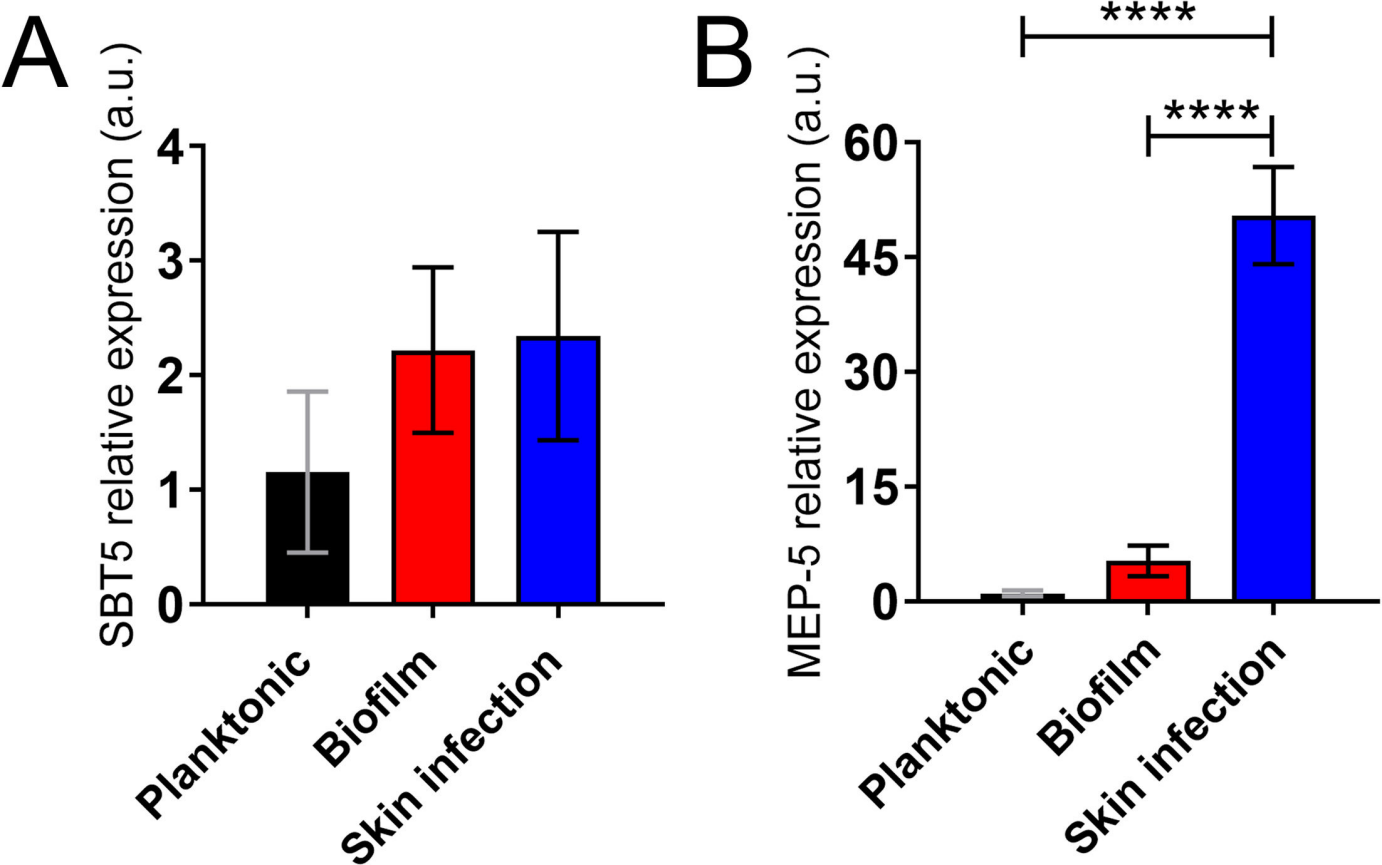
Relative expression of (**A**) *Sbt5* and (**B**) *Mep5* in *T. rubrum* in planktonic form, biofilm, and during the 3D skin model infection. Bars represent the mean values, and error bars denote standard deviations. Significance (*P* < 0.0001***) was determined by one-way ANOVA and adjusted using Tukey’s post-hoc analysis.

Through qPCR, the relative expression of interest for *T. rubrum* genes was assessed in various conditions. The expression of the *Sbt5* gene, which encodes for subtilisin 5 and, although not significant among the groups, was onefold higher for fungal biofilms *in vitro* in the RHS model compared to the planktonic cells (Fig. 9A). Notably, the expression of the *Mep5* gene (Fig. 9B), which encodes an extracellular MEP-5, was 5.09 and 48.5 times higher for the *T. rubrum* biofilm-derived cells *in vitro* and RHS infected models, respectively, relative to planktonic cells (*P* < 0.0001). Increased expression of *Mep5* by *T. rubrum* may indicate the importance of MEP-5 in host-dermatophyte interactions, particularly in biofilm formation. Furthermore, these results highlight the differential expression of genes by *T. rubrum*, which can be influenced by the fungal phenotype (e.g., biofilm versus planktonic) and the specific infection model.

## DISCUSSION

Dermatophytes are fungi that affect keratinized tissues, such as skin, initiating infections at the epidermal barrier and leading to dysfunctions of the stratum corneum and cornified skin appendages, which cause dermatophytosis. These infections remain relatively understudied, particularly in terms of the role of biofilms and the challenges in obtaining suitable models for studying them. Indeed, cells cultured as monolayers do not undergo the keratinization process required to study the adherence and invasion of dermatophytes (13, 53, 54).

Therefore, the present study investigated the biofilm formation of *T. rubrum* in 3D models using skin tissue or spheroids by CM, SEM, and relative gene expression analysis via real-time PCR. These models create an environment suitable for the growth of dermatophyte biofilms, with nutritional characteristics like those of *in vivo* conditions.

Biofilms are considered an important virulence factor, and the nutritional substrate available for biofilm growth can interfere with the production of the extracellular polymeric matrix, which in turn decreases biofilm permeability. The formation of biofilms, sessile, multicellular fungi surrounded by a protective extracellular matrix, allows fungi to evade current antifungal therapies and contribute to the observed antifungal resistance, as well as the high recurrence rates of dermatophytosis (36, 55–58). In this work, the biofilms formed by the *T. rubrum* strain showed optical density values comparable to those reported by Costa-Orlandi et al. (32), indicating a high-quality biofilm formation with no difference in either biomass formation or metabolic activity observed between the two media used (*P* > 0.05) as described before (32). In recent years, there has been a growing appreciation for the role of biofilms in human medicine. However, in some mycotic diseases, there is still a need to prove biofilms in *in vivo* or in *ex vivo* models. Several models have been used to study fungal skin infections, such as reconstructed human epidermis (51, 52). Reconstructed human epidermis and full-thickness skin models are valuable tools for research in dermatology, toxicology, and cosmetics. The *ex vivo* model with human skin is ideal for simulating skin infections, as it realistically presents the three-dimensional structure of the tissue (8, 50, 59–61). Both models, however, lack the complexity of a more robust model, such as organoids, capable of incorporating various cell lines, making it possible to produce immune response, a crucial component of fungal colonization, which the absence may have contributed to a more pronounced invasion in deeper layers of the 3D model (62). Given that a gap in research exists regarding the use of new models to study fungal-host interactions, the RHS model is well suited for investigating fungal colonization and invasion, two crucial processes in dermatophytosis, allowing further investigations in the future. Then, our study aimed to elucidate biofilm formation; thus, we chose to focus on 4-day infections to examine the biofilm formation despite the impaired epidermal barrier. The photomicrograph of the RHS skin model was also included. From histological sections, it can be observed that the stratum corneum is well formed, and all layers are visible. Some studies report that by the fourth day post-infection with *T. rubrum*, reconstructed human epidermis models exhibit significant tissue damage and deeper fungal invasion (13) using arthroconidia. Our findings are similar to those obtained in previous manuscripts.

Porcine skin explants also show intensive damage when infected with *T. rubrum*. Ho et al. (63) demonstrated through histopathological analysis that porcine skin explants infected with *T. rubrum* conidia and incubated for 72 h exhibited marked damage and degradation of the epidermis, along with evident fungal invasion into the dermis.

In our work, both models revealed characteristic structures of biofilms, where a dense network of elongated hyphae surrounded by adhered extracellular matrix formed after infection with conidia (31, 32). This finding demonstrates the biofilm-forming capacity of *T. rubrum* in *ex vivo* models under conditions that emulate those found in the clinic, as observed *in vitro* (31). This may explain the persistent infection, resistance, and the need for long-term treatment required for dermatophytosis (36, 64).

Culture models in the form of spheroids have also emerged, offering a quick and cost-effective method (47). CM of human keratinocyte spheroids demonstrated a high index of cell survival during the analysis period. Cell survivability was maintained, even after *T. rubrum* infection, resulting in an extensive network of hyphae throughout the entire surface of the 3D models. CM images revealed the formation of fungal structures beneath the spheroid, indicating that keratinocytes and *T. rubrum* cells colocalized on the same slices of the image, demonstrating the model as a promising option for investigating *T. rubrum* infection and interaction in a 3D environment (50). We verified that the hypha appears much thinner when compared to *in vitro* and *ex vivo* biofilm, with differences in hyphal morphology and organization within the extracellular matrix. *In vitro*, hyphae can grow in various directions, while the extracellular matrix fills the spaces between them. In *ex vivo* conditions, fungal elements and matrix can replace the natural surface in some areas, resulting in a more compact biofilm. This observation was verified by other researchers (55, 57).

Endoproteases are crucial to dermatophyte fungi, which are responsible for fungal colonization and the degradation of keratinized tissues during infection. Dermatophytes produce numerous proteases, including collagenolytic, elastolytic enzymes, and keratinases (65). The prominent families of proteases secreted by fungi are subtilisins (serine proteases) and fungalysins (metalloproteases). Studies on *T. rubrum* have shown that metalloproteases and serine proteinases are potential virulence-related factors in dermatophytes (66–68). Still, these proteases have not been studied in biofilm and RHS infection with *T. rubrum*. In this study, we tested the expression of two endoproteases, subtilisin and MEP-5, in both planktonic and biofilm forms *in vitro*, as well as during *T. rubrum* infection of an RHS model. Notably, Mep5 was significantly expressed on *T. rubrum-*derived cells grown on the RHS model relative to those formed *in vitro* biofilm and planktonic cells. Similar to our SEM images, *T. rubrum* has demonstrated the capacity for biofilm formation under *ex vivo* conditions. *T. rubrum* is a highly specialized pathogen with evolutionary adaptations that enable it to thrive on human skin. The adaptation enables the fungus, unlike nearly all other fungal species, to grow to a certain extent in the nutritionally poor environment of the skin (28) and express its virulence factors, such as MEP-5, which is essential for tissue invasion. Given its significant expression during infection, MEP-5 is a promising therapeutic target for developing novel antifungal drugs to manage dermatophytosis. Endoproteases produced by dermatophytes during *in vivo* infection differ from those made by the same dermatophytes *in vitro* (11, 69), thereby increasing the importance of studying virulence factors under conditions as close as possible to *in vivo* infection to analyze the pathogenesis of dermatophytosis accurately. Future comparative studies using *ex vivo* and *in vivo* models of *T. rubrum* infection are warranted to validate similarities in infection and virulence factor expression and dissect the potential differences or limitations of the *ex vivo* models.

In conclusion, these findings position 3D models as valuable tools for advancing the understanding of host-pathogen interactions, ultimately contributing to pathogenesis and maybe to the development of targeted treatments for chronic fungal infections.
